# Single- Versus Double-Layer Uterine Closure After Cesarean Section Delivery: A Systematic Review and Meta-Analysis

**DOI:** 10.7759/cureus.18405

**Published:** 2021-09-30

**Authors:** Kaif Qayum, Irfan Kar, Junaid Sofi, Hari Panneerselvam

**Affiliations:** 1 General Surgery, Wye Valley NHS Foundation Trust, Hereford, GBR; 2 General Surgery, Sher-I-Kashmir Institute of Medical Sciences, Srinagar, IND; 3 Medicine, Wye Valley NHS Foundation Trust, Hereford, GBR

**Keywords:** double-layer, single-layer, uterine closure, residual myometrium thickness, cesarean section

## Abstract

Cesarean section (CS) delivery is a common procedure, and its incidence is increasing globally. To compare single-layer (SL) with double-layer (DL) uterine closure techniques after cesarean section in terms of ultrasonographic findings and rate of CS complications. PubMed, Scopus, Web of Science, and Cochrane Library were searched for relevant randomized clinical trials (RCTs). Retrieved articles were screened, and relevant studies were included in a meta-analysis. Continuous data were pooled as mean difference (MD) with 95% confidence interval (CI), and dichotomous data were pooled as relative risk (RR) and 95% CI. Analysis was conducted using RevMan software (Version 5.4). Eighteen RCTs were included in our study. Pooled results favored DL uterine closure in terms of residual myometrial thickness (MD = -1.15; 95% CI -1.69, -0.60; P < 0.0001) and dysmenorrhea (RR = 1.36; 95% CI 1.02, 1.81; P = 0.04), while SL closure had shorter operation time than DL closure (MD = -2.25; 95% CI -3.29, -1.21; P < 0.00001). Both techniques had similar results in terms of uterine dehiscence or rupture (RR = 1.88; 95% CI 0.63, 5.62; P = 0.26), healing ratio (MD = -5.00; 95% CI -12.40, 2.39; P = 0.18), maternal infectious morbidity (RR = 0.94; 95% CI 0.66, 1.34; P = 0.72), hospital stay (MD = -0.12; 95% CI -0.30, 0.06; P = 0.18), and readmission rate (RR = 0.95; 95% CI 0.64, 1.40; P = 0.78). Double-layer uterine closure shows more residual myometrial thickness and lower incidence of dysmenorrhea than single-layer uterine closure of cesarean section scar. But single-layer closure has the advantage of the shorter operation time. Both methods have comparable blood loss amount, healing ratio, hospital stay duration, maternal infection risk, readmission rate, and uterine dehiscence or rupture risk.

## Introduction and background

Cesarean section (CS) incidence is increasing globally through recent years, reaching 25% of total deliveries in some countries [1]. This rise in the incidence of CS increases the events of CS-related complications [2]. CS complications include infection, hemorrhage and thromboembolism as short-term complications in addition to the long-term complications and symptoms including dysmenorrhea, dysuria, abnormal uterine bleeding, and infertility [2,3]. Some CS complications − such as placenta accreta, uterine rupture or dehiscence, and CS scar pregnancy − may be manifested during a subsequent pregnancy due to a defective uterine scar [3]. The prevalence of uterine scar defect in women with previous CS is unexpectedly high, ranging from 56% to 84% when examined by transvaginal ultrasonography with contrast [4]. 

In pregnant women with a previous cesarean delivery, the risk of uterine rupture during a subsequent trial of labor has to be assessed. Its assessment is done using ultrasonographic measurement of the lower uterine segment and the residual myometrial thickness (RMT) [5]. Defective RMT was linked to a higher risk of adverse outcomes, including postmenstrual spotting, uterine dehiscence or rupture, placental adherence, failure of labor trial, and more complications of CS scar pregnancy [6]. It has been hypothesized that uterine incision closure technique may be associated with the development of the uterine niche and subsequent CS-related adverse outcomes [4].

The surgical method of uterine closure after CS is suggested to affect the RMT, uterine scar defect, and the healing of the uterine scar. However, clear evidence of the best method for uterine closure is not established [7], and no evidence-based guideline for the closure technique is present [8]. Previous studies reported that double-layer (DL) closure has thicker residual myometrium and a lower incidence of large defects than single-layer (SL) closure. However, a clear conclusion about other clinical outcomes is still lacking [9]. 

This systematic review and meta-analysis aim to compare the ultrasonographic findings and complication rate of single-layer (SL) versus double-layer (DL) uterine closure techniques after CS procedure.

## Review

Methods

This systematic review and meta-analysis followed the steps described in the "Preferred Reporting Items for Systematic reviews and Meta-Analyses (PRISMA)" and in the "Cochrane handbook for systematic reviews of interventions" [10,11].

Data Collection and Search Strategy

We searched PubMed, Scopus, Web of Science, and Cochrane library databases for published randomized control trials (RCTs) from inception till June 2021. We used the following keywords: "surgical technique," "endometrium," "suture technique," "single-layer," "double-layer," "cesarean section," and "postcesarean." We applied no restrictions regarding age, publication date, the indication of cesarean section, or the number of previous deliveries.

Inclusion and Exclusion Criteria

We included RCTs that compared SL versus DL uterine closure techniques after cesarean section delivery and reported any of the ultrasonographic outcomes or adverse events. We excluded observational studies, reviews, non-randomized trials, cross-sectional studies, editorials, abstracts, thesis, letters, books, and chapters.

Screening and Study Selection

Retrieved records were imported to Endnote software, and duplicates were removed. The remaining records underwent title and abstract screening then full-text screening according to our eligibility criteria. Three reviewers performed the screening process independently, and any disagreement was solved by discussion. Eligible articles were included in the meta-analysis.

Data Extraction

All study authors shared in the data extraction. We extracted data related to the following domains: (1) summary of the included studies, including the study name, national clinical trial (NCT) number registration number, country where the study was carried out, sample size, period of follow-up, and study outcomes, (2) baseline characters of the included studies' population, including study arms, age of the participant, gestational age at delivery, body mass index, birth weight, nulliparity, preterm delivery, multiple pregnancy, elective cesarean delivery, and prior cesarean deliveries, (3) outcomes, including residual myometrial thickness, dysmenorrhea, uterine dehiscence or rupture, healing ratio, blood loss, operative time, maternal infectious morbidity, hospital stay, and readmission rate, and (4) quality assessment domains.

Quality Assessment

According to the Cochrane Collaboration tool for risk of bias assessment in randomized studies, we evaluated the quality of the included studies [12]. The tool included the judgment of the selection, performance, detection, attrition, reporting, and other bias domains. Each domain was judged as low, high, or unclear risk of bias. At least two independent reviewers judged each domain and conflicts were solved by discussion.

Statistical Analysis

Data analysis was conducted using review manager (RevMan) software version 5.4. Data of continuous outcomes were reported as mean difference (MD) and 95% confidence interval (CI) using the Inverse-Variance method, and dichotomous data were reported as relative risk (RR) and 95% CI using the Mantel-Haenszel method. We assessed heterogeneity using chi-square and I-square tests, and heterogeneity was considered significant at chi-square P-value < 0.1 and I2 > 50%. We used the random-effects model for analysis. Whenever pooled data are heterogeneous, we tried to solve the heterogeneity by sensitivity analysis using the leave-one-out test and subgroup analysis. We performed a subgroup analysis, when applicable, according to the used suturing technique, whether locked or unlocked sutures, and whether the decidua is included or excluded from suturing.

Results

Literature Search and Study Selection

**Figure 1:**
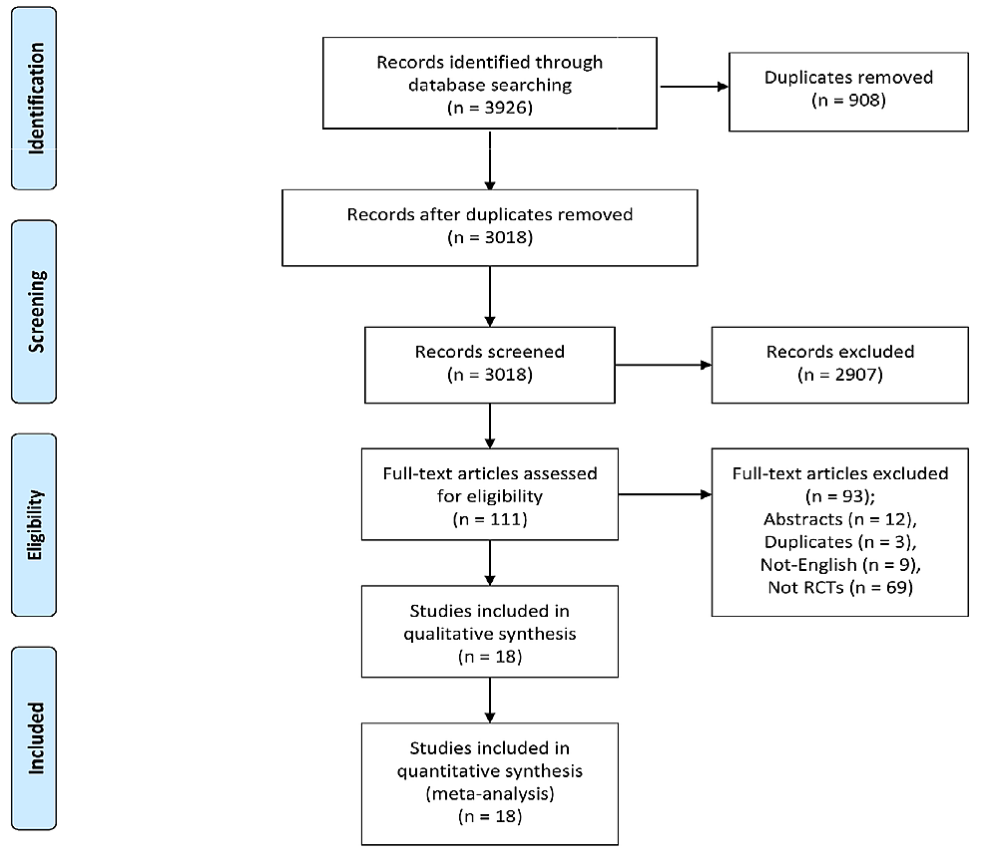
PRISMA flow chart summarizing the process of data collection and study selection. PRISMA: Preferred Reporting Items for Systematic reviews and Meta-Analyses.

Searching electronic databases yielded a total of 3926 articles. After removing duplicates, we had 3018 unique articles that underwent title and abstract screening. Of these articles, 2907 were excluded, and 111 full-texts were retrieved and screened according to our eligibility criteria. Finally, 18 studies were considered eligible for inclusion in the meta-analysis [3,8,13-28]. Figure 1 summarizes the flow of the study selection process and data collection.

Characteristics of the Included Studies

Included studies were performed in various countries. The sample size varied considerably across studies, ranging from 30 to 7411. The follow-up period varied from six weeks in some studies to 6-24 months in other studies. The mean age of included patient groups ranged from 24 to 32 years, while mean gestational age ranged from 37.8 to 40 weeks. Table 1 and Table 2 show the summary of included studies and the baseline characters of included patients, respectively.

**Table 1:** Summary of the included studies. W, weeks; M, months; Y, years; NCT, National Clinical Trial; ISRCTN, International Standard Randomized Controlled Trial; OXTREC, Oxford Tropical Research Ethics Committee.

ID	Country	NCT	Sample size	Follow-up	Outcomes
Bamberg 2016 [14]	Germany	NCT 02338388	306	6-24 M	Residual myometrium thickness, blood loss, operative time, maternal infectious morbidity
Bennich 2016 [15]	Denmark	NCT02144805	76	5 M	Residual myometrium thickness, dysmenorrhea, healing ratio, blood loss, operative time
CAESAR 2010 [13]	Multicenter	ISRCTN 11849611	2979	6 W	Operative time, maternal infectious morbidity, hospital stay, readmission rate
Chapman 1997 [16]	United States	-	145	4 Y	Uterine rupture, hospital stay
CORONIS 2016 [3]	International	OXTREC; 013-06a	7411	3 Y	Dysmenorrhea, uterine rupture
El-Gharib 2013 [18]	Egypt	-	150	6 W	Residual myometrium thickness, operative time, maternal infectious morbidity, hospital stay
Hamar 2007 [19]	United States	NCT00224250	30	6 W	Residual myometrium thickness, blood loss, operative time
Hanacek 2019 [20]	Czech Republic	-	540	12 M	Residual myometrium thickness, maternal infectious morbidity
Hauth 1992 [21]	United States	-	906	-	Maternal infectious morbidity
Kalem 2019 [22]	Turkey	-	138	-	Residual myometrium thickness, dysmenorrhea, operative time
Khamees 2018 [17]	Egypt	-	80	-	Residual myometrium thickness, blood loss, operative time
Roberge 2016 [24]	Canada	NCT01860859	54	6-12 M	Residual myometrium thickness, healing ratio, blood loss, operative time, maternal infectious morbidity
Sevket 2014 [25]	Turkey	-	36	6 M	Residual myometrium thickness, healing ratio, blood loss, operative time
Shrestha 2015 [26]	Nepal	-	50	6 W	Residual myometrium thickness
Sood 2005 [23]	India	-	208	6 W	Blood loss, operative time, maternal infectious morbidity, hospital stay
Stegwee 2020 [8]	Netherlands	2015.462	2852	9 M	Residual myometrium thickness, healing ratio, blood loss, operative time, hospital stay, readmission rate
Yasmin 2011 [27]	Pakistan	-	60	6 W	Residual myometrium thickness, uterine rupture, blood loss, operative time
Yilmazbaran 2020 [28]	Turkey	NCT03629028	282	6-9 M	Residual myometrium thickness, dysmenorrhea, operative time

**Table 2:** Baseline characteristics of the included studies' population. Data are presented as mean ± standard deviation, median (interquartile range), or number (percentage). *Range. SL, single-layer uterine closure; DL, double-layer uterine closure.

ID	Arms	Number	Age, year	Gestational age at delivery, week	Body mass index, kg/m^2^	Birthweight, kg	Nulliparity	Preterm delivery	Multiples	Elective cesarean	Prior cesarean deliveries
Bamberg 2016 [14]	SL	149	31.8 ± 5.6	37.8 ± 2.2	26.1 ± 5.7	3.15 ± 0.75	63 (42 %)	28 (19 %)	16 (11 %)	117 (78 %)	55 (37%)
	DL	129	30.3 ± 6.5	37.3 ± 2.3	25.6 ± 6.2	3.09 ± 0.67	48 (37 %)	25 (19 %)	13 (10 %)	103 (79 %)	57 (44%)
Bennich 2016 [15]	SL	35	30.3 ± 4.5	38.7 ± 0.6	24.6±4.8	-	35 (47.9)	-	-	35 (47.9)	-
	DL	38	30.5 ± 5.5	38.9 ± 0.7	24.1±3.5	-	38 (52.1)	-	-	38 (52.1)	-
CAESAR 2010 [13]	SL	1483	30.6 ± 5.9	39.0 ± 2.0	-	-	989 (67)	-	79	-	-
	DL	1496	30.6 ± 5.9	39.1 ± 1.9	-	-	480 (32)	-	76	-	-
Chapman 1997 [16]	SL	70	-	37 ± 5.2	-	-	-	14	-	-	-
	DL	75	-	40 ± 3.7	-	-	-	25	-	-	-
CORONIS 2016 [3]	SL	4705	-	-	-	-	-	-	-	-	-
	DL	4711	-	-	-	-	-	-	-	-	-
El-Gharib 2013 [18]	SL	75	28.84 ± 3.4	39.11 ± 0.7	-	2.86 ± 0.6	75 (100%)	-	-	-	0 (0%)
	DL	75	28.36 ± 3.2	39.16 ± 0.7	-	2.87 ± 0.6	75 (100%)	-	-	-	0 (0%)
Hamar 2007 [19]	SL	15	30 ± 7	39.3 ± 0.5	-	3.35 ± 0.75	11 (73%)	-	-	-	-
	DL	15	25 ± 7	38.6 ± 0.9	-	3.44 ± 0.43	8 (53%)	-	-	-	-
Hanacek 2019 [20]	SL	149	31 (29-34)	40 (39-41)	22.4 (20.4-25.3)	-	-	-		-	-
	DL	175	32 (29-34)	40 (40-41)	22.3 (20.1-24.2)	-	-	-		-	-
Hauth 1992 [21]	SL	457	24.2	38	-	-	220 (48%)	-	16 (6%)	-	126 (28%)
	DL	449	24.6	37.8	-	-	239 (53%)	-	20 (4%)	-	99 (22%)
Kalem 2019 [22]	SL	68	29.25 ± 6.27	38.5 ± 2.7	26.04 ± 2.37	3.23 ± 0.51	-	-	-	-	-
	DL	70	28.94 ± 5. 17	39.4 ± 3.6	25.90 ± 2.28	3.26 ± 0.49	-	-	-	-	-
Khamees 2018 [17]	SL	40	-	-	-	-	40 (100%)	-	-	-	0 (0%)
	DL	40	-	-	-	-	40 (100%)	-	-	-	0 (0%)
Roberge 2016 [24]	SL	27	30.8 ± 4.0	39.2 ± 0.6	25.1 ± 4.7	3.35 ± 0.379	22	-	-	-	-
	DL with locked sutures	27	31.1 ± 6.4	39.1 ± 0.5	23.5 ± 3.9	3.41 ± 0.44	20	-	-	-	-
	DL with unlocked sutures	27	31 ± 3.7	38.9 ± 0.6	25.1 ± 5.3	3.24 ± 0.47	20	-	-	-	-
Sevket 2014 [25]	SL	15	29.7 ± 6.5	38.6 ± 0.8	-	3.44 ± 0.43		-	-	-	-
	DL	16	29.4 ± 7.3	39 ± 1.2	-	3.39 ± 0.38		-	-	-	-
Shrestha 2015 [26]	SL	25	26.04 ± 5.06	38.36 ± 2.21	-	-	21 (84%)	-	-	16 (64%)	-
	DL	25	23.92 ± 4.32	38.92 ± 1.35	-	-	17 (68%)	-	-	8 (32%)	-
Sood 2005 [23]	SL	102	26.5 ± 4.5	38.2 ± 1.5	-	-	-	-	-	66 (64.7%)	34 (33.4%)
	DL	106	25.4 ± 3.5	37.8 ± 1.8	-	-	-	-	-	75 (70.7%)	37 (35%)
Stegwee 2020 [8]	SL	1144	32 ± 4.7	-	26.4 ± 4.6	-	-	-	80 (7%)	-	-
	DL	1148	32.1 ± 4.6	-	26.6 ± 4.8	-	-	-	91 (7.9%)	-	-
Yasmin 2011 [27]	SL	30	20-35*	37-40*	-	-	-	-	-	-	30 (100%)
	DL	30	20-35*	37-40*	-	-	-	-	-	-	30 (100%)
Yilmazbaran 2020 [28]	SL	109	29.8 ± 4.1	38 ± 2	28.9 ± 4.2	3.19 ± 0.57	103 (94.5)	-	-	79 (72%)	-
	DL	116	30.8 ± 5.1	38.1 ± 2.1	29.8 ± 4.6	3.26 ± 0.63	105 (91.3)	-	-	81 (69.3%)	-

Quality Assessment

**Figure 2:**
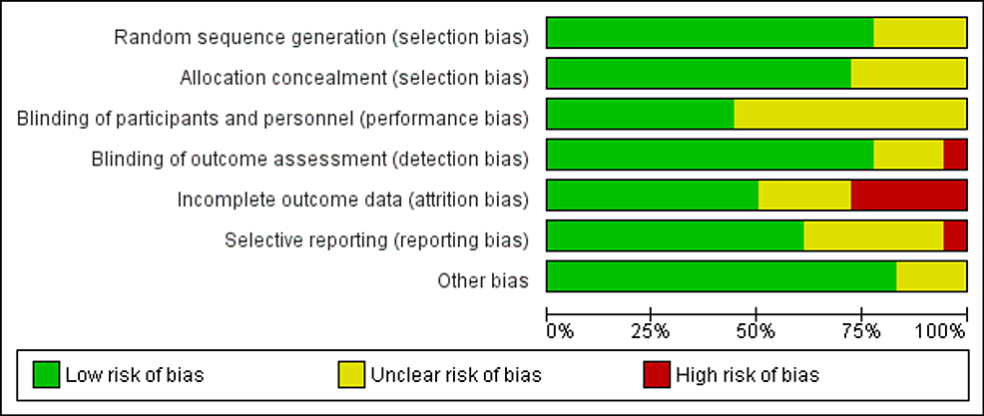
Risk of bias graph.

**Figure 3:**
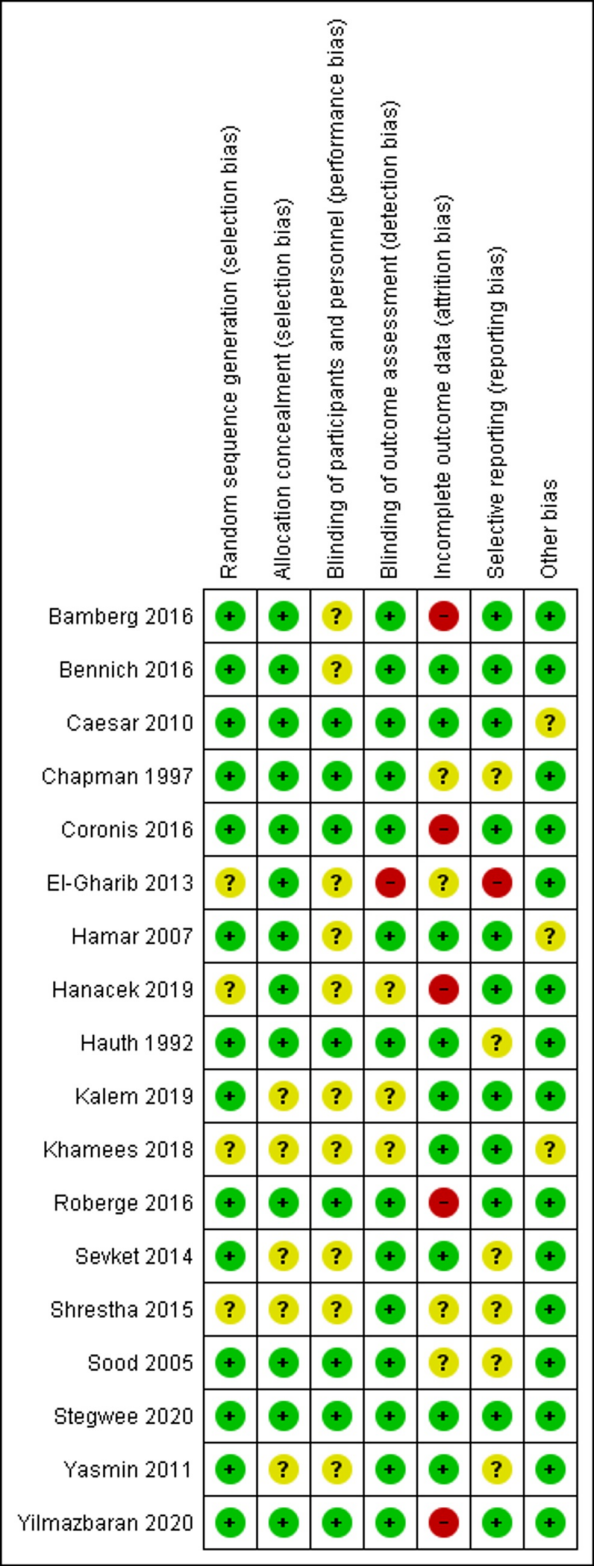
Risk of bias summary.

Most included studies had a low risk of selection bias regarding both selection bias domains: random sequence generation and allocation concealment. However, the remaining studies were of unclear risk of selection bias because the reported data are insufficient to judge. Most studies had an unclear risk of performance bias because they reported scarce details to judge the blinding process of participants and personnel. In contrast, detection bias was at low risk in most studies due to proper blinding of the outcome assessor. Attrition bias was at low risk in most studies because the lost data are insufficient to produce bias results. Reporting bias was judged low risk in most studies because the outcomes of interest were reported as expected. The "other bias" domain was judged low risk in most studies and unclear in some studies. The risk of bias graph shows the overall judgment of each risk of bias domain (Figure 2) and the risk of bias summary summarizes the judgment of each domain in each study (Figure 3).

Outcomes

**Figure 4:**
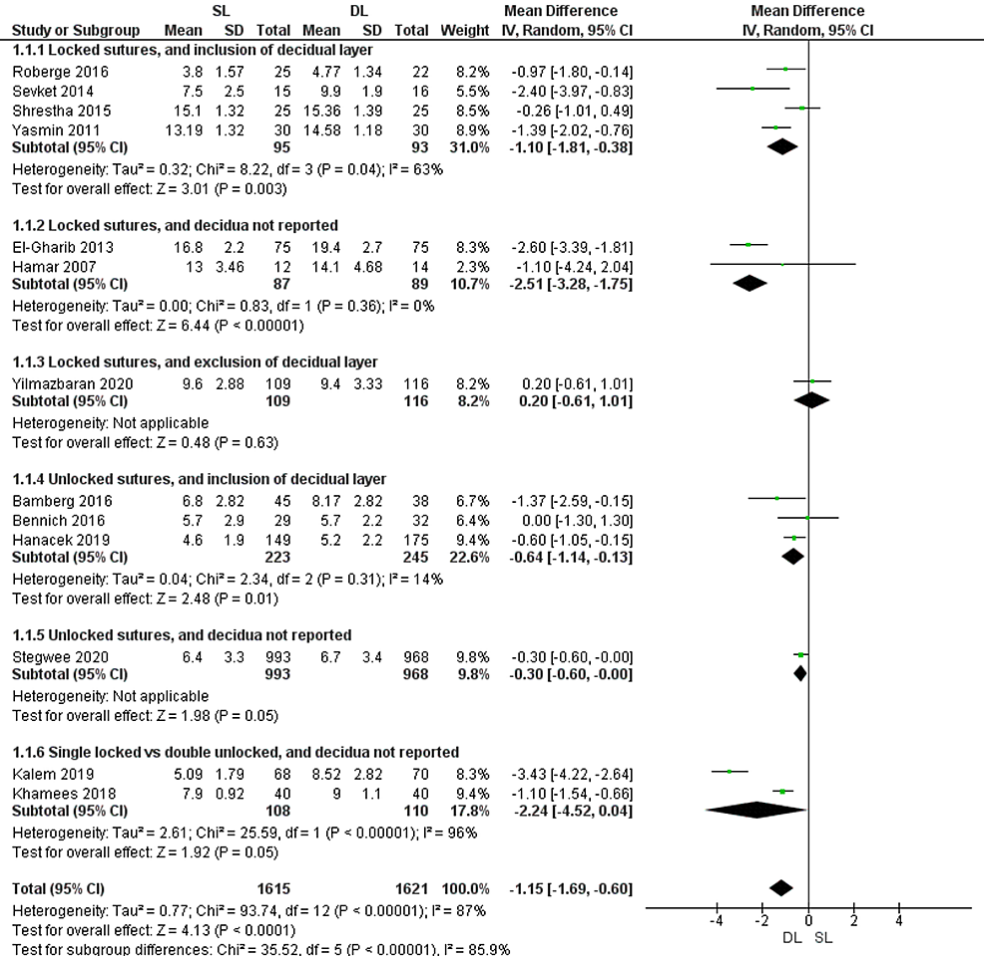
Forest plot comparing single- versus double-layer uterine closure in terms of residual myometrial thickness. SL, single-layer uterine closure; DL, double-layer uterine closure.

Residual myometrial thickness (Figure 4)

The residual myometrial thickness was significantly lower with the SL compared with the DL uterine closure technique (MD = -1.15; 95% CI -1.69, -0.60; P < 0.0001). Pooled data are heterogeneous (P < 0.00001; I2 = 88%). In the subgroup of locked sutures with inclusion of the decidua, SL uterine closure showed lower RMT (MD = -1.10; 95% CI -1.81, -0.38; P = 0.003) and the results were heterogeneous, but heterogeneity was solved after excluding Shrestha 2015 and the results remained significant. Pooled results were also lower with SL than DL uterine closure in the subgroup of locked sutures with no data about decidual layer inclusion (MD = -2.51; 95% CI -3.28, -1.75; P < 0.00001), and the results were homogeneous (P = 0.36; I2 = 0%). Also, in the subgroup of unlocked sutures with inclusion of the decidua, SL showed lower RMT than DL uterine closure (MD = -0.64; 95% CI -1.14, -0.13; P = 0.01) and the results were homogeneous (P = 0.31; I2 = 14%). Also, the subgroup analysis comparing SL closure using locked sutures versus DL closure using unlocked sutures showed no significant difference between both groups (MD = -2.24; 95% CI -4.52, 0.04; P = 0.05) and the results were heterogeneous (P < 0.00001; I = 96%).

**Figure 5:**
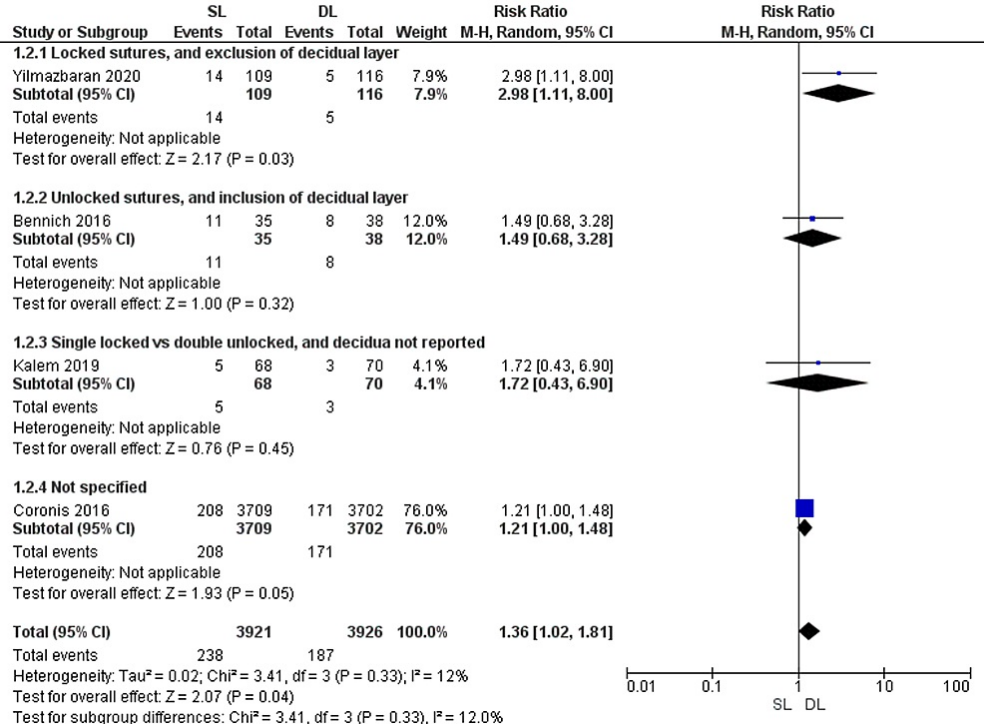
Forest plot comparing single- versus double-layer uterine closure in terms of dysmenorrhea. SL, single-layer uterine closure; DL, double-layer uterine closure.

Dysmenorrhea (Figure 5)

Pooled data showed higher risk of dysmenorrhea with SL than DL uterine closure (RR = 1.36; 95% CI 1.02, 1.81; P = 0.04).Pooled data were homogeneous (P = 0.33; I2 = 12%).

**Figure 6:**
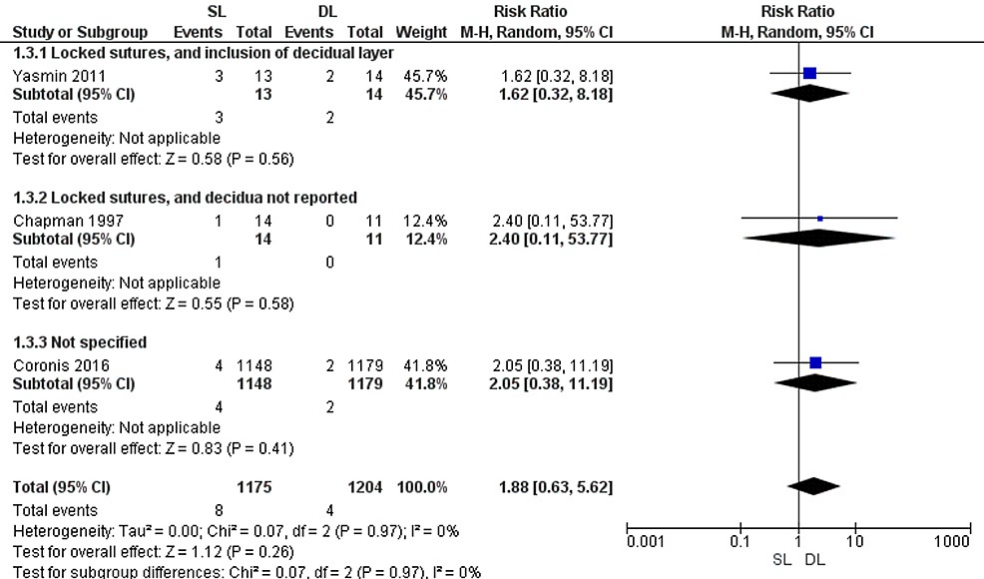
Forest plot comparing single- versus double-layer uterine closure in terms of uterine dehiscence or rupture. SL, single-layer uterine closure; DL, double-layer uterine closure.

Uterine dehiscence or rupture (Figure 6)

The risk of uterine dehiscence or rupture was similar with SL and DL uterine closure (RR = 1.88; 95% CI 0.63, 5.62; P = 0.26). Pooled results were homogeneous (P = 0.97; I2 = 0%).

**Figure 7:**
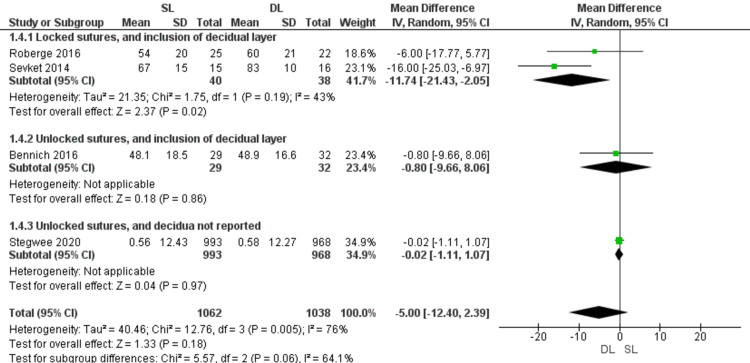
Forest plot comparing single- versus double-layer uterine closure in terms of healing ratio. SL, single-layer uterine closure; DL, double-layer uterine closure.

Healing ratio (Figure 7)

Healing ratio was comparable with SL and DL uterine closure (MD = -5.00; 95% CI -12.40, 2.39; P = 0.18). Pooled data were heterogeneous (P = 0.005, I2 = 76%). However, SL uterine closure showed lower healing ratio in the subgroup of locked sutures with inclusion of the decidual layer (MD = -11.74; 95% CI -21.43, -2.05; P = 0.02), and pooled data were homogeneous (P = 0.19; I2 = 43%).

**Figure 8:**
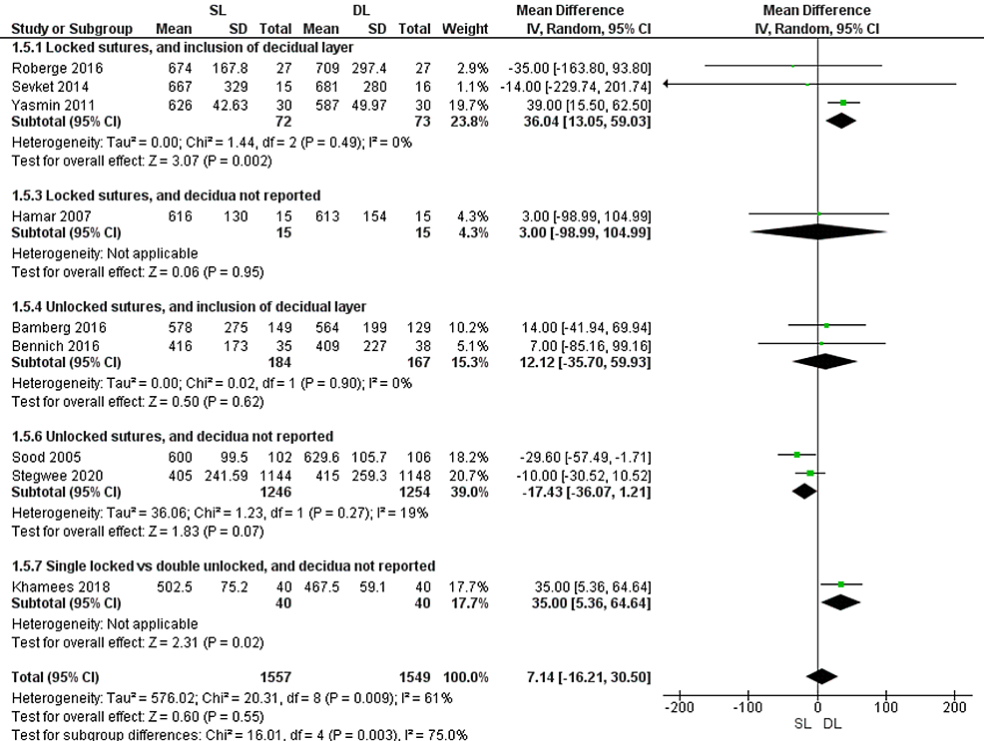
Forest plot comparing single- versus double-layer uterine closure in terms of blood loss. SL, single-layer uterine closure; DL, double-layer uterine closure.

Blood loss (Figure 8)

Pooled data showed that the amount of blood loss was comparable with SL and DL uterine closure (MD = 7.14; 95% CI -16.21, 30.50; P = 0.55). Pooled results were heterogeneous (P = 0.009; I2 = 61%). However, the subgroup analysis of patients who had locked sutures with inclusion of the decidua favored DL over SL uterine closure in the amount of blood loss (MD = 36.04; 95% CI 13.05, 59.03; P = 0.002), and the data were homogeneous (P = 0.49; I2 = 0%). The subgroup who had unlocked sutures with inclusion of the decidual layer showed insignificant results (MD = 12.12; 95% CI -35.70, 59.93; P = 0.62), and data were homogeneous (P = 0.90; I2 = 0%). Also, the subgroup of unlocked sutures with no data about including the decidua showed no significant difference between SL and DL closure techniques (MD = -17.43; 95% CI -36,07, 1.21; P = 0.07), and the results were homogeneous (P = 0.27; I2 = 19%).

**Figure 9:**
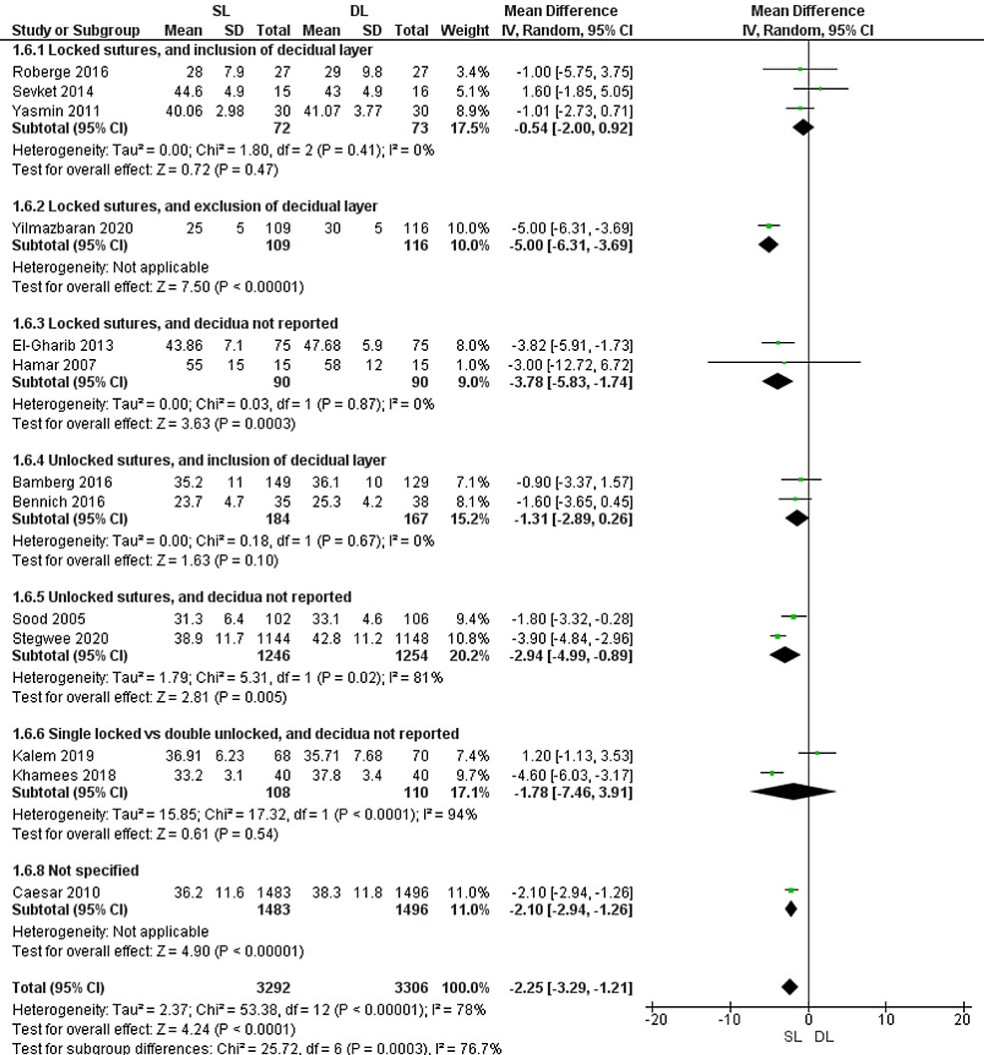
Forest plot comparing single- versus double-layer uterine closure in terms of operative time. SL, single-layer uterine closure; DL, double-layer uterine closure.

Operative time (Figure 9)

Pooled data showed that operative time is shorter with SL than with DL uterine closure (MD = -2.25; 95% CI -3.29, -1.21; P < 0.00001). Pooled results were heterogeneous (P < 0.00001; I2 = 78%). Similar results were observed in the subgroup of locked sutures with no data about including the decidua (MD = -3.78; 95% CI -5.83, -1.74; P = 0.0003) (homogeneous data, P = 0.87; I2 = 0%), and in the subgroup of unlocked sutures with no data about including the decidua (MD = -2.94; 95% CI -4.99, -0.89; P = 0.005) (heterogeneous data, P = 0.02; I2 = 94%). The difference between SL and DL closure was insignificant in the subgroup of unlocked sutures with inclusion of the decidua (MD = -1.31; 95% CI -2.89, 0.26; P = 0.1) (homogeneous data, P = 0.67; I2= 0%), and in the subgroup comparing SL closure using locked sutures versus DL closure using unlocked sutures (MD = -1.78; 95% CI -7.46, 3.91; P = 0.54) (heterogeneous data, P < 0.0001; I2 = 94%).

**Figure 10:**
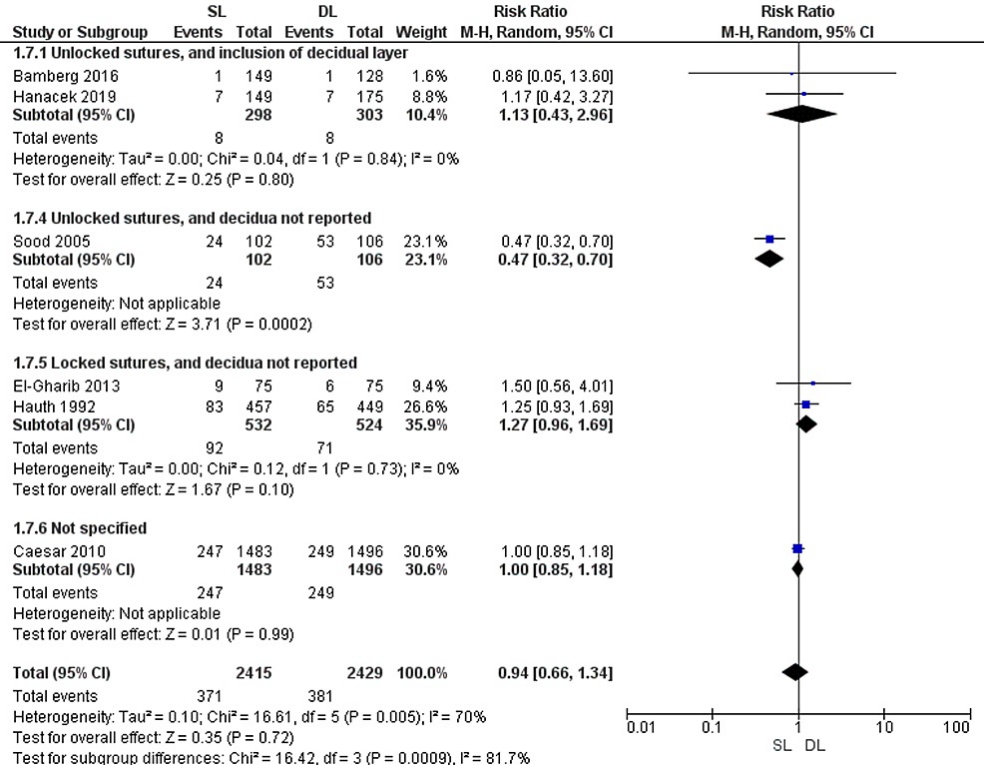
Forest plot comparing single- versus double-layer uterine closure in terms of maternal infectious morbidity. SL, single-layer uterine closure; DL, double-layer uterine closure.

Maternal infectious morbidity (Figure 10)

Pooled data showed no significant difference between SL and DL uterine closure in the risk of maternal infection morbidity (RR = 0.94; 95% CI 0.66, 1.34; P = 0.72). Pooled results are heterogeneous (P = 0.005, I2 = 70%). Also, the difference was insignificant in the subgroups of unlocked sutures including the decidua (RR = 1.13; 95% CI 0.43, 2.96; P = 0.8) (homogeneous data, P = 0.84; I2 = 0%) and the subgroup of locked sutures with no data about including the decidua (RR = 1.27; 95% CI 0.96, 1.69; P = 0.1) (homogeneous data, P = 0.73, I2 = 0%).

**Figure 11:**
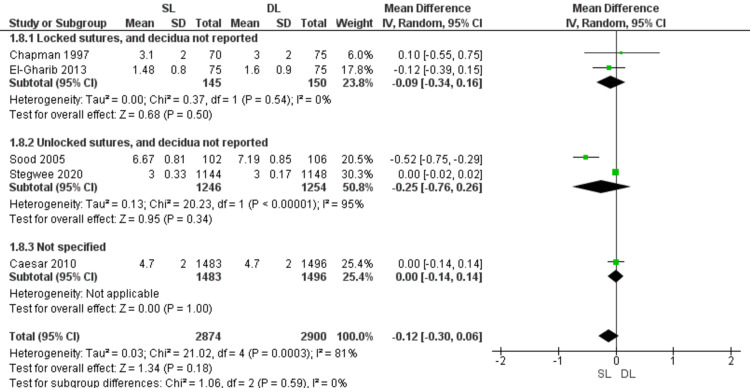
Forest plot comparing single- versus double-layer uterine closure in terms of hospital stay. SL, single-layer uterine closure; DL, double-layer uterine closure.

Hospital stay (Figure 11)

Pooled data showed no significant difference between SL and DL uterine closure in the period of hospital stay after procedure (MD = -0.12; 95% CI -0.30, 0.06; P = 0.18). Pooled results are heterogeneous (P = 0.0003: I2 = 81%). Also, the difference was insignificant in the subgroups of locked sutures with no data about including the decidua (MD = -0.09; 95% CI -0.34, 0.16; P = 0.5) (homogeneous data, P = 0.54; I2 = 0%) and the subgroup of unlocked sutures with no data about including the decidua (MD = -0.25; 95% CI -0.76, 0.26; P = 0.34) (heterogeneous data, P < 0.00001, I2 = 95%).

**Figure 12:**
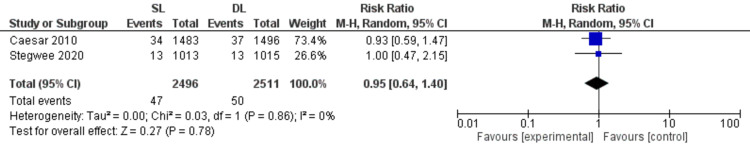
Forest plot comparing single- versus double-layer uterine closure in terms of readmission rate. SL, single-layer uterine closure; DL, double-layer uterine closure.

Readmission rate (Figure 12)

Pooled data showed similar risk of readmission rate with SL and DL uterine closure techniques (RR = 0.95; 95% CI 0.64, 1.40; P = 0.78). Pooled results are homogeneous (P = 0.86, I2 = 0%).

Discussion

The analysis of 19 RCTs' results revealed that DL uterine closure is better than SL uterine closure after cesarean delivery in RMT and dysmenorrhea. Both techniques showed comparable results about the amount of blood loss, healing ratio, hospital stay duration, maternal infection risk, readmission rate, and the risk of uterine dehiscence or rupture during a subsequent delivery. In contrast, SL closure showed better results regarding operative time.

As reported by previous studies [7,9], SL uterine closure was associated with thinner RMT than the DL closure technique. This finding was more evident in our study when using locked sutures in both SL and DL closure methods. Also, a previous meta-analysis reported superiority of DL closure with unlocked sutures over SL closure with locked sutures regarding RMT [9]. We performed a similar comparison in a subgroup analysis, but the pooled estimate did not reach statistical significance. The thicker RMT with DL uterine exposure is expected due to the separate closure of the myometrial and serosal layers in the DL technique.

Previous studies support our finding that SL closure is associated with more risk of dysmenorrhea than DL closure [3, 9, 15]. However, a recent trial reported similar rates with both techniques [8]. In our study, the superiority of the DL closure method was attributed to the recent trial by Yilmaz Baran 2020 [28], while the other pooled trials showed insignificant results [3,15,22]. Thus, the present study solves this debate in favor of the DL closure technique by pooling the results of all previously published RCTs. The higher risk of dysmenorrhea with DL closure has no obvious cause.

Regarding the risk of uterine dehiscence or rupture during the following pregnancy, our results coincide with the literature that both SL and DL closure techniques have comparable risks [7,9,29].

Our study addressed other short- and long-term outcomes. These included the amount of blood loss, the duration of hospital stay post-procedure, the readmission rate, and the maternal infection rate. These outcomes' pooled results showed no significant difference between SL and DL closure. Similar findings were reported by a previous meta-analysis [9].

Stegwee 2018 meta-analysis reported that the healing ratio is better with DL closure than SL closure [9]. Our results showed no significant difference between both groups. This disagreement mostly arises from data from observational studies included in the previous meta-analysis [9] but not in the present study.

As the results of our study found, SL closure is known to be easier and faster than DL closure [9,28]. Most obstetricians prefer SL to DL closure in order to decrease operative time with no significant increase in the risk of complications [3,13]. Also, a recent randomized multi-center study stated that SL closure is associated with lower niche prevalence, less need for treatment of gynecological complications, and less harmful effect on sexual activity and general health [8].

Although the DL closure showed better sonographic outcomes, as revealed by the present study and previous studies [9], these outcomes seem to be clinically insignificant [8,28]. Thus, SL closure is still the most popular method for uterine closure after CS delivery.

It is important to name an optimal standard method for uterine closure after CS. This is because cesarean delivery is a popular procedure, exceeding one million cases in the United States per year [30]. Also, this would help the decision-making for pregnant women who had a previous cesarean delivery, whether they will undergo a trial of labor or an elective repeat cesarean delivery [30].

In this systematic review and meta-analysis, we included RCTs only to provide high-quality class-one evidence and followed the widely accepted PRISMA guidelines during the conduction of this study. We included all published RCTs with no publication date restriction. In addition, we performed subgroup analyses according to variations in the surgical techniques (locked or unlocked sutures, and inclusion or exclusion of the decidua) to solve the heterogeneity between studies. Limitations in this study include the heterogeneity detected in many outcomes and could not be solved in some cases. In addition, some long-term outcomes were reported by a small number of studies, which limits the generalizability of the results. Future studies with large sample size and longer follow-up would provide more conclusive results.

## Conclusions

DL uterine closure technique was associated with more RMT compared with SL closure technique. Also, patients who had DL uterine closure showed lower incidence of dysmenorrhea. On the other hand, SL closure was associated with significantly shorter operation time. Both techniques showed comparable healing ratio, readmission rate, and hospital stay. Also, the amount of blood loss, the risk of maternal infection, and the risk of uterine dehiscence or rupture during a subsequent delivery were similar with both techniques.

## References

[1] Boerma T, Ronsmans C, Melesse DY (2018). Global epidemiology of use of and disparities in caesarean sections. Lancet.

[2] Sandall J, Tribe RM, Avery L (2018). Short-term and long-term effects of caesarean section on the health of women and children. Lancet.

[3] Abalos E, Addo V, Brocklehurst P (2016). Caesarean section surgical techniques: 3 year follow-up of the CORONIS fractional, factorial, unmasked, randomised controlled trial. Lancet.

[4] Bij de Vaate AJ, van der Voet LF, Naji O (2014). Prevalence, potential risk factors for development and symptoms related to the presence of uterine niches following Cesarean section: systematic review. Ultrasound Obstet Gynecol.

[5] Kok N, Wiersma IC, Opmeer BC, de Graaf IM, Mol BW, Pajkrt E (2013). Sonographic measurement of lower uterine segment thickness to predict uterine rupture during a trial of labor in women with previous Cesarean section: a meta-analysis. Ultrasound Obstet Gynecol.

[6] van der Voet LF, Bij de Vaate AM, Veersema S, Brölmann HA, Huirne JA (2014). Long-term complications of caesarean section. The niche in the scar: a prospective cohort study on niche prevalence and its relation to abnormal uterine bleeding. BJOG.

[7] Roberge S, Demers S, Berghella V, Chaillet N, Moore L, Bujold E (2014). Impact of single- vs double-layer closure on adverse outcomes and uterine scar defect: a systematic review and metaanalysis. Am J Obstet Gynecol.

[8] Stegwee SI, van der Voet LF, Ben AJ (2021). Effect of single- versus double-layer uterine closure during caesarean section on postmenstrual spotting (2Close): multicentre, double-blind, randomised controlled superiority trial. BJOG.

[9] Stegwee SI, Jordans I, van der Voet LF (2018). Uterine caesarean closure techniques affect ultrasound findings and maternal outcomes: a systematic review and meta-analysis. BJOG.

[10] Higgins JP, Thomas J, Chandler J, Cumpston M, Li T, Page MJ, Welch VA (2019). Cochrane Handbook for Systematic Reviews of Interventions. 2nd Edition.

[11] Liberati A, Altman DG, Tetzlaff J (2009). The PRISMA statement for reporting systematic reviews and meta-analyses of studies that evaluate health care interventions: explanation and elaboration. PLoS Med.

[12] Higgins JP, Altman DG, Gøtzsche PC (2011). The Cochrane Collaboration's tool for assessing risk of bias in randomised trials. BMJ.

[13] The CAESAR study collaborative group (2010). Caesarean section surgical techniques: a randomised factorial trial (CAESAR). BJOG.

[14] Bamberg C, Dudenhausen JW, Bujak V (2018). A prospective randomized clinical trial of single vs. double layer closure of hysterotomy at the time of cesarean delivery: the effect on uterine scar thickness. Ultraschall Med.

[15] Bennich G, Rudnicki M, Wilken-Jensen C, Lousen T, Lassen PD, Wøjdemann K (2016). Impact of adding a second layer to a single unlocked closure of a Cesarean uterine incision: randomized controlled trial. Ultrasound Obstet Gynecol.

[16] Chapman SJ, Owen J, Hauth JC (1997). One- versus two-layer closure of a low transverse cesarean: the next pregnancy. Obstet Gynecol.

[17] Khamees RE, Khedr AH, Shaaban M, Bahi-Eldin M (2018). Effect of single versus double layer suturing on healing of uterine scar after cesarean delivery. Suez Canal Univ Med J.

[18] ELGharib MN, Awara AM (2013). Ultrasound evaluation of the uterine scar thickness after single versus double layer closure of transverse lower segment cesarean section. J Basic Clin Reprod Sci.

[19] Hamar BD, Saber SB, Cackovic M (2007). Ultrasound evaluation of the uterine scar after cesarean delivery: a randomized controlled trial of one- and two-layer closure. Obstet Gynecol.

[20] Hanacek J, Vojtech J, Urbankova I, Krcmar M, Křepelka P, Feyereisl J, Krofta L (2020). Ultrasound cesarean scar assessment one year postpartum in relation to one- or two-layer uterine suture closure. Acta Obstet Gynecol Scand.

[21] Hauth JC, Owen J, Davis RO (1992). Transverse uterine incision closure: one versus two layers. Am J Obstet Gynecol.

[22] Kalem Z, Kaya AE, Bakırarar B, Basbug A, Kalem MN (2021). An optimal uterine closure technique for better scar healing and avoiding isthmocele in cesarean section: a randomized controlled study. J Invest Surg.

[23] Kumar SA (2005). Single versus double layer closure of low transverse uterine incision at cesarean section. J Obstet Gynecol India.

[24] Roberge S, Demers S, Girard M (2016). Impact of uterine closure on residual myometrial thickness after cesarean: a randomized controlled trial. Am J Obstet Gynecol.

[25] Sevket O, Ates S, Molla T, Ozkal F, Uysal O, Dansuk R (2014). Hydrosonographic assessment of the effects of 2 different suturing techniques on healing of the uterine scar after cesarean delivery. Int J Gynaecol Obstet.

[26] Shrestha P, Shrestha S, Gyawali M (2015). Ultrasound evaluation of uterine scar in primary caesarean section: a study of single versus double layer uterine closure. Am J Public Health Res.

[27] Yasmin S, Sadaf J, Fatima N (2011). Impact of methods for uterine incision closure on repeat caesarean section scar of lower uterine segment. J Coll Physicians Surg Pak.

[28] Yılmaz Baran Ş, Kalaycı H, Doğan Durdağ G, Yetkinel S, Alemdaroğlu S, Çok T, Bulgan Kılıçdağ E (2021). Single- or double-layer uterine closure techniques following cesarean: A randomized trial. Acta Obstet Gynecol Scand.

[29] Hesselman S, Högberg U, Ekholm-Selling K, Råssjö EB, Jonsson M (2015). The risk of uterine rupture is not increased with single- compared with double-layer closure: a Swedish cohort study. BJOG.

[30] Menacker F, Declercq E, Macdorman MF (2006). Cesarean delivery: background, trends, and epidemiology. Semin Perinatol.

